# Detection of endo-epicardial atrial low-voltage areas using unipolar and omnipolar voltage mapping

**DOI:** 10.3389/fphys.2022.1030025

**Published:** 2022-10-06

**Authors:** Mathijs S. Van Schie, Paul Knops, Lu Zhang, Frank R. N. Van Schaagen, Yannick J. H. J. Taverne, Natasja M. S. De Groot

**Affiliations:** ^1^ Department of Cardiology, Erasmus Medical Center, Rotterdam, Netherlands; ^2^ Department of Cardiothoracic Surgery, Erasmus Medical Center, Rotterdam, Netherlands

**Keywords:** voltage mapping, unipolar voltage, omnipolar mapping, low-voltage areas, endo-epicardial mapping, atrial fibillation, sinus rhythm

## Abstract

**Background:** Low-voltage areas (LVA) can be located exclusively at either the endocardium or epicardium. This has only been demonstrated for bipolar voltages, but the value of unipolar and omnipolar voltages recorded from either the endocardium and epicardium in predicting LVAs at the opposite layer remains unknown. The goal of this study was therefore to compare simultaneously recorded endo-epicardial unipolar and omnipolar potentials and to determine whether their voltage characteristics are predictive for opposite LVAs.

**Methods:** Intra-operative simultaneous endo-epicardial mapping (256 electrodes, interelectrode distances 2 mm) was performed during sinus rhythm at the right atrium in 93 patients (67 ± 9 years, 73 male). Cliques of four electrodes (2 × 2 mm) were used to define maximal omnipolar (V_omni,max_) and unipolar (V_uni,max_) voltages. LVAs were defined as V_omni,max_ ≤0.5 mV or V_uni,max_ ≤1.0 mV.

**Results:** The majority of both unipolar and omnipolar LVAs were located at only the endocardium (74.2% and 82.0% respectively) or epicardium (52.7% and 47.6% respectively). Of the endocardial unipolar LVAs, 25.8% were also located at the opposite layer and 47.3% vice-versa. In omnipolar LVAs, 18.0% of the endocardial LVAs were also located at the epicardium and 52.4% vice-versa. The combination of epicardial V_uni,max_ and V_omni,max_ was most accurate in identifying dual-layer LVAs (50.4%).

**Conclusion:** Unipolar and omnipolar LVAs are frequently located exclusively at either the endocardium or epicardium. Endo-epicardial LVAs are most accurately identified using combined epicardial unipolar and omnipolar voltages. Therefore, a combined endo-epicardial unipolar and omnipolar mapping approach is favoured as it may be more indicative of possible arrhythmogenic substrates.

## Introduction

Endocardial bipolar voltage mapping has emerged as an invasive tool for defining potential target sites for ablation therapy of atrial tachyarrhythmia such as atrial fibrillation (AF). Low-voltage areas (LVA) are commonly considered surrogate markers for areas of conduction disorders, which play a key role in perpetuation of AF (Kottkamp et al., 2015; Knops et al., 2016). However, the efficacy of such bipolar voltage-guided ablation strategies remains controversial. Especially in patients with persistent AF, the presence of intramural or epicardial substrate limits the efficacy of endocardial ablation strategies (De Martino et al., 2021). Recently, Piorkowski et al. (2018)) showed that bipolar LVAs can also be located exclusively at either the endocardium or epicardium, therefore favoring an endo-epicardial ablation approach.

We demonstrated that identification of LVAs was considerably directional dependent when using bipolar voltages (van Schie et al., 2021a). Bipolar LVAs could also contain large unipolar voltages. In addition, there were not solely conduction disorders within LVAs, but also high conduction velocities (CV). It could therefore be that the bipolar low-voltage threshold overestimates the size of dense scar and still harbors islets and channels of viable tissue (Chopra et al., 2014).

Endocardial bipolar recordings are still mainly used to detect scar tissue areas as it represents more local activity. In the ventricles it has been demonstrated that unipolar voltage mapping is preferred to identify intramural or epicardial substrates as unipolar electrograms (EGM) comprise a larger region of myocardial electrical activity (Sim et al., 2019). On the other hand, omnipolar voltage mapping can be used to extract maximal bipolar voltage from a collection of EGMs in order to minimize directional differences of bipolar voltages. A combination of unipolar and omnipolar voltage may therefore provide additional information on the underlying tissue and it may also be more indicative of transmural substrates (Chopra et al., 2014; van Schie et al., 2021a).

It, however, remains unknown whether simultaneously recorded atrial endo- and epicardial unipolar and omnipolar voltages are complementary or contradictory on identifying LVAs at the opposite layer. We therefore performed simultaneously endo-epicardial high-density mapping to 1) examine endo- and epicardial characteristics of unipolar and omnipolar voltages, 2) explore the relation between various types of voltages in identification of LVAs from an endocardial and epicardial point of view, and 3) examine whether characteristics of LVAs can be predictive for LVAs at the opposite layer.

## Materials and methods

### Study population

The study population consisted of 93 successive adult patients undergoing elective open heart coronary artery bypass grafting, aortic or mitral valve surgery or a combination of valvular surgery and bypass grafting in the Erasmus Medical Center Rotterdam. This study was approved by the institutional medical ethical committee (MEC 2015-373). Written informed consent was obtained from all patients and patient characteristics (e.g., age, medical history and cardiovascular risk factors) were obtained from the patient’s medical record.

### Simultaneous endo-epicardial mapping procedure

**FIGURE 1:**
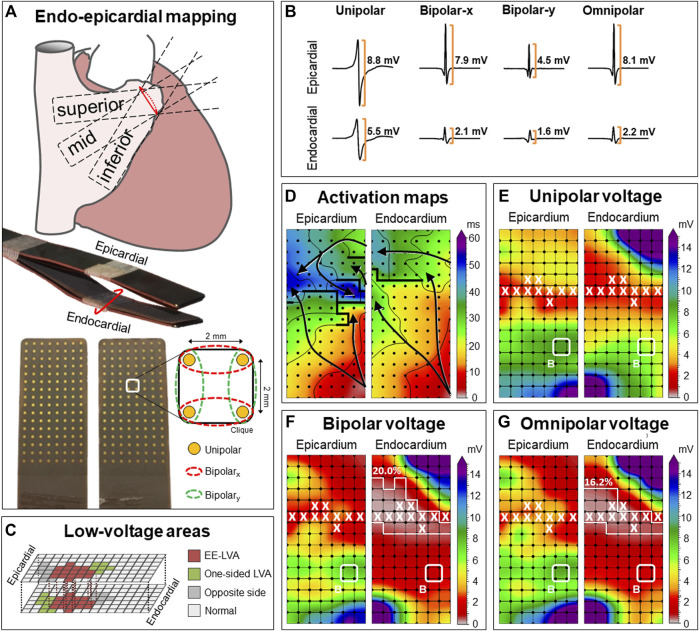
Construction of unipolar, bipolar and omnipolar voltages in 2 × 2 mm cliques. **(A)** Two high-density electrode arrays consisting of 128 unipolar electrodes are fixed together. One array was placed on the epicardium and one array is introduced into the RA using the incision for venous cannulation to map the endocardium and epicardium simultaneously. The RA was mapped with the tip of the electrode arrays toward the inferior vena cava (inferior), the superior vena cava (superior) and in between, toward the terminal crest (mid). For each square area, enclosed by four electrodes, four unipolar EGMs and matched bipolar and omnipolar EGMs were derived from two electrode orientations [along the vertical *y*-axis (green) and horizontal *x*-axis (red)] as indicated by the dotted lines. **(B)** Examples of a unipolar, horizontal bipolar-x, vertical bipolar-y and omnipolar EGM recorded from both the epicardium (upper) and endocardium (lower). The two bipolar EGMs differed considerably, illustrating the electrode orientation dependence of bipolar mapping. Omnipolar mapping provides electrode orientation-independent voltages that are larger than the bipolar with the largest measurable peak-to-peak voltage, in both cases the horizontal bipolar-x EGMs. **(C)** LVAs can be either located at solely the endocardium or epicardium (green) or at both sides (red). The corresponding clique at the exact opposite side is highlighted (grey). The other cliques are then indicated as normal voltage (light grey). **(D)** Example of endo-epicardial activation time maps with isochrones drawn at every 10 ms. Arrows indicate the main direction of the propagation wavefront and thick black lines indicate areas of conduction block (time difference between adjacent electrodes ≥12 ms). **(E–G)** Peak-to-peak voltages of corresponding EGMs are used to create different voltage maps. Bipolar voltage map illustrates the maximal bipolar voltage in both horizontal and vertical orientations within one clique. LVAs are highlighted by a white line and areas of EEA are indicated by a white X. In this example, endocardial bipolar and omnipolar LVA are present in respectively 20.0% and 16.2% of the cliques. EEA was present in 9.5% of the cliques at both the endocardium and epicardium. EEA = endo-epicardial asynchrony; EGM = electrogram; (EE-) LVA = (endo-epicardial-) low-voltage area.

An overview of the methodology is provided in Figure 1A and previously described in detail (Knops et al., 2016). Two electrode arrays, each containing 128 (8 × 16) unipolar electrodes with a diameter of 0.45 mm and 2 mm interelectrode spacing, were secured on two bendable spatulas and were located on the exact opposite side of each other. A temporally bipolar epicardial pacemaker wire attached to the right atrial (RA) free wall served as a reference electrode and the indifferent electrode consisted of a steel wire fixed to subcutaneous tissue of the thoracic cavity. Simultaneous endo- and epicardial mapping was performed after heparinization and arterial cannulation but before commencement of extracorporeal circulation. One spatula (marked as the endocardial electrode array) was introduced in the RA after incising the RA appendage and closed with the purse-string suture. To prevent overlap of recording areas near the right atrial incision, the endocardial electrode array was introduced into the RA for at least 1.5 cm extra after introducing the last row of electrodes. Unipolar EGMs were recorded for 5 s during stable sinus rhythm at the superior, middle and inferior RA free wall (Figure 1A), including a surface ECG lead I, a calibration signal of 2 mV and 1,000 ms and a bipolar reference EGM. Data were stored on a hard disk after amplification (gain 1,000), filtering (bandwidth 0.5–400 Hz), sampling (1 kHz) and analogue to digital conversion (16 bits).

### Omnipolar voltage mapping

In order to create omnipolar EGMs, bipolar EGMs were first created by subtracting two neighboring unipolar EGMs in horizontal (bipolar-x) and vertical direction (bipolar-y) and subsequently filtered (bandwidth 30–400 Hz) as demonstrated in Figure 1A. Omnipolar EGMs were then created from these bipolar EGMs as previously described (Deno et al., 2017; van Schie et al., 2021a). Within a square area defined by four adjacent electrodes (a *clique*), omnipolar EGMs were used to mathematically obtain bipolar EGMs in any direction without physically rotating the sensing electrodes of the bipolar pair. Within a clique, a 2-dimensional voltage vector $\vec{v}\left(t\right)$ is derived from an electric field of a passing activation wavefront from which the maximal extent of two orthogonal bipolar EGMs is calculated over the interval ( T ) containing one SR beat (Haldar et al., 2017):

$$V_{\max}=\max_{t_{i},t_{j}\in T}\left\{\left|\vec{v}\left(t_{i}\right)-\vec{v}\left(t_{j}\right)\right|\right\}$$

$V_{\max}$ corresponds to the peak-to-peak amplitude of a bipolar voltage signal obtained along the unit vector direction $\hat{m}$ where $t_{i}$ and $t_{j}$ are now the times associated with $V_{\max}$ in which $t_{i}>t_{j}$

$$\hat{m}=\frac{\vec{v}\left(t_{i}\right)-\vec{v}\left(t_{j}\right)}{V_{\max}}$$

$V_{\max}$ provides an objective measure of the largest possible bipolar EGM within a clique without the ambiguity of electrode orientation and is used to describe omnipolar EGM voltages.

### Data analysis

Unipolar and omnipolar EGMs were semi-automatically analyzed using custom-made software. The steepest negative slope of a unipolar atrial potential was marked as the local activation time (LAT), providing that the amplitude of the deflection was at least two times the estimated noise level of the unipolar EGM (de Groot et al., 2016). In case of fractionated potentials, the deflection with the steepest slope was taken as LAT. All annotations were manually checked with a consensus of two investigators. CV was computed at each electrode from LATs using discrete velocity vectors (DVV) as previously described (van Schie et al., 2021b). The DVV method uses all eight neighboring electrodes to compute an average local propagation velocity for the center electrode. Endo-epicardial LAT differences were determined by selecting the median of the LAT differences within the exact opposite electrode and its eight surrounding electrodes (Kharbanda et al., 2020). Potential voltage was defined as the peak-to-peak amplitude of the steepest deflection (unipolar) or highest peak (omnipolar) as demonstrated in Figure 1B.

As omnipolar EGMs can only be derived in square areas, unipolar potentials were correlated to each other in areas of 2 × 2 mm -a *clique*- which contain four unipolar EGMs and the corresponding omnipolar EGMs (Figure 1A). Subsequently, the maximal potential voltage of the unipolar and omnipolar EGMs pertaining to that area were computed, resulting in two values (V_uni,max_ and V_omni,max_). In addition, the mean of the magnitudes of the four CV estimates derived from the four unipolar LATs was used as indication of the CV within the 2 × 2 mm area. Areas corresponding to a mean CV of 0 cm/s were excluded to avoid inclusion of far field potentials. To calculate endo-epicardial asynchrony (EEA), the minimum of endo-epicardial LAT differences within a clique was taken. EEA was then defined as a transmural difference in electrical activation of ≥15 ms (de Groot et al., 2016). As a unipolar voltage cut-off of ≤1.0 mV and bipolar voltage cut-off of ≤0.5 mV are most frequently used in daily clinical practice to identify LVAs, we also used these values as the “golden standard” to identify low-voltage cliques (Sanders et al., 2003; Kharbanda et al., 2021; van Schie et al., 2021c). Endo-epicardial LVAs (EE-LVA) are defined as LVA cliques located at both the endocardium and epicardial at the exact same site (Figure 1C).

### Statistical analysis

Normally distributed data are expressed as mean ± SD, whereas skewed data are expressed as median [25th–75th percentile]. Clinical characteristics were compared using Student’s *t*-test or Mann-Whitney *U* test when appropriate. Categorical data are expressed as numbers (percentages) and analyzed with a χ2 or Fisher exact test. Paired voltage and velocity data were analyzed between the endo- and epicardium using a paired *t*-test or Wilcoxon signed-rank test. Correlation was determined by ordinary least squares regression. A *p*-value <0.05 was considered statistically significant.

## Results

### Study population

Clinical characteristics of the study population [*N* = 93, age 67 (61–72) years, 73 male (78.5%)] are summarized in Table 1. A history of AF was present in 37 (39.8%) patients. Ischemic- or valvular heart disease or combined heart diseases were present in 47 (50.5%), 24 (25.8%), and 21 (22.6%) patients respectively. Most patients used class II antiarrhythmic drugs (*N* = 64, 68.8%).

**TABLE 1:** Baseline characteristics.

	
Patients	93
Male	73 (78.5%)
Age (years)	67 [61–72]
BMI (kg/m^2^)	28.0 [24.6–31.1]
Underlying heart disease	
- iHD	47 (50.5%)
- vHD	24 (25.8%)
- cHD	21 (22.6%)
History of AF	37 (39.8%)
- Paroxysmal	31 (33.3%)
- Persistent	4 (4.3%)
- Longstanding persistent	2 (2.2%)
Cardiovascular risk factors	
Hypertension	56 (60.2%)
Diabetes mellitus	32 (34.4%)
Hypercholesterolemia	50 (53.8%)
Left ventricular ejection fraction <40%	12 (12.9%)
Antiarrhythmic drugs	
- Class I	1 (1.1%)
- Class II	64 (68.8%)
- Class III	6 (6.5%)
- Class IV	7 (7.5%)

Values are presented as N (%) or median [interquartile ranges].

BMI, body mass index; iHD, ischemic heart disease; vHD, valvular heart disease; cHD, combined heart disease; AF, atrial fibrillation; LVEF, left ventricular ejection fraction.

### Clique characteristics

In the entire study population, a total of 281 mapping locations resulted in 406,571 unipolar and 723,695 bipolar potentials from which 164,704 cliques were created for both the endocardium and epicardium (329,408 cliques in total; 3,542 ± 1,610 per patient). The mean CV of each clique was 83.6 [62.3–103.4] cm/s at the endocardium and 84.4 [67.0–100.5] cm/s at the epicardium (*p* < 0.001, *R* ^2^ = 0.853, $CV_{epi}=0.91∙CV_{endo}$ ). EEA was present in 2.9% of all cliques. An example of differences in activation and voltage maps constructed by using unipolar and corresponding bipolar and omnipolar EGMs are illustrated in Figures 1D–G.

### Relation between endo-and epicardial voltages

**FIGURE 2:**
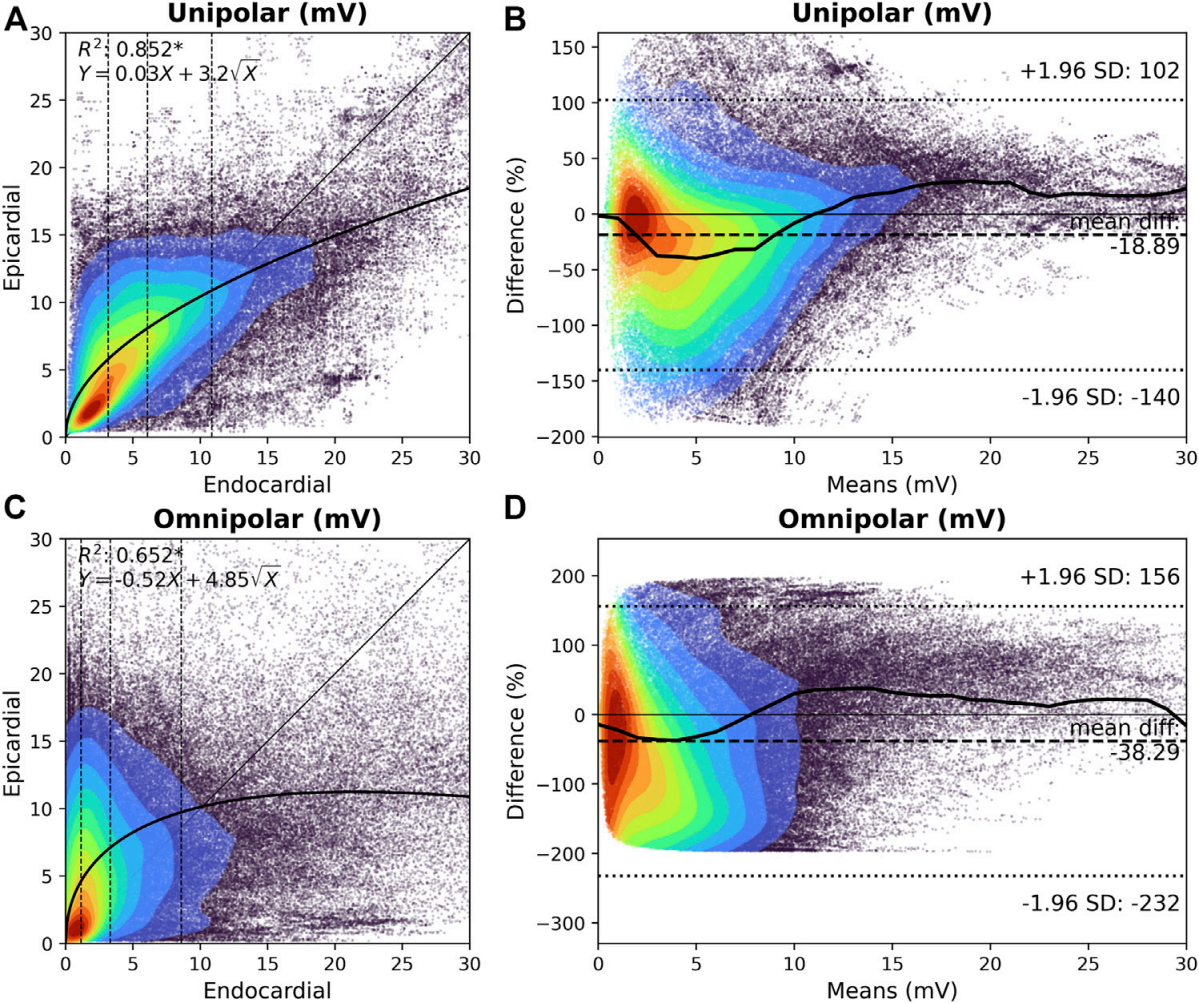
Relation between endocardial and epicardial unipolar and omnipolar voltage. **(A)** and **(C)** Kernel density plots of V_uni,max_ **(A)** and V_omni,max_ **(C)** voltages between the endocardium and epicardium. The colors indicate the data density. A black line indicates the ordinary least squares prediction. Statistical significance is indicated by an asterisk (*p* < 0.001). V_uni,max_ and V_omni,max_ are subdivided according to the 25th, 50th, and 75th percentiles of the endocardial voltages, and are indicated by dashed vertical lines. **(B)** and **(D)** Bland-Altman plots of endocardial *versus* epicardial V_uni,max_ **(B)** and V_omni,max_ **(D)** voltages. The colors indicate the data density. A black line indicates the median per one means mV. The mean difference and 95% confidence intervals are indicated by the dashed lines. V_omni,max_ = omnipolar clique voltage; V_uni,max_ = unipolar clique voltage.

The upper panel of Figure 2 demonstrates the relationship between endocardial and the corresponding epicardial V_uni,max_. In 61% of the cliques, V_uni,max_ at the epicardium was larger than the corresponding V_uni,max_ at the endocardium (8.2 [4.9–11.6] mV vs. 6.1 [3.2–10.9] mV, *p* < 0.001). When V_uni,max_ was subdivided into four quartiles, V_uni,max_ at the epicardium was especially larger at the lower V_uni,max_ values at the endocardium (p0-p25: 83.7%; p25-p50: 79.8%; p50-p75: 61.7%; p75-p100: 18.6%). As a consequence, there was a strong inversely quadratic relation with linear component (*R* ^2^ = 0.852, $Y=0.03X+3.2\sqrt{X}$ ) between V_uni,max_ at the endocardium and epicardium. As illustrated in Figure 2B, V_uni,max_ up to 12 mV were particularly larger at the epicardium than the endocardium, although there was a large variation in the differences. Epicardial V_uni,max_ were on average 19% larger than endocardial V_uni,max_.

The lower panel of Figure 2 demonstrates the relationship between endocardial and the corresponding epicardial V_omni,max_. In 64% of the cliques, V_omni,max_ at the epicardium was larger compared to the corresponding endocardial cliques (6.7 [3.2–10.9] mV vs. 3.3 [1.1–8.6] mV, *p* < 0.001, respectively). When V_omni,max_ was subdivided into four quartiles, V_omni,max_ at the epicardium was especially larger at the lower V_omni,max_ values at the endocardium (p0-p25: 91.3%; p25-p50: 86.5%; p50-p75: 62.2%; p75-p100: 15.5%). However, there was no clear relationship between the endocardial and epicardial V_omni,max_, although lower endocardial voltages were associated with a larger range of epicardial voltages. As illustrated in Figure 2D, V_omni,max_ up to 8 mV were particularly larger at the epicardium than the endocardium. Epicardial V_omni,max_ were on average 38% larger than endocardial V_omni,max_.

### Endo-epicardial differences in low-voltage areas

Characteristics of unipolar and omnipolar LVAs at the endo- and epicardium are listed in Table 2. Endocardial and epicardial unipolar LVAs were present at respectively 3.1% and 1.7% of the mapping area. At the endo- and epicardium, unipolar LVAs corresponded to respectively 86.8% and 73.3% of the omnipolar LVA.

**TABLE 2:** Characteristics of low-voltage areas.

	Unipolar		Omnipolar	
	Normal	LVA (≤1.0 mV)	Normal	LVA (≤0.5 mV)
Endocardial	96.9%	3.1%	88.3%	11.7%
Unipolar (mV)	6.3 [3.4–11.1]	0.8 [0.6–0.9]	6.9 [4.1–11.8]	1.5 [1.1–2.0]
Omnipolar (mV)	3.5 [1.3–8.9]	0.2 [0.2–0.4]	4.2 [1.7–9.7]	0.3 [0.2–0.4]
CV (cm/s)	84.3 [63.3–103.8]	52.0 [28.6–79.2]	86.3 [66.4–105.2]	55.7 [34.2–80.6]
EEA (%)	2.4%	18.3%	2.1%	8.8%
Epicardial	98.3%	1.7%	96.0%	4.0%
Unipolar (mV)	8.3 [5.1–11.7]	0.8 [0.7–0.9]	8.5 [5.3–11.8]	1.3 [0.9–1.8]
Omnipolar (mV)	6.8 [3.4–11.0]	0.3 [0.2–0.5]	7.0 [3.6–11.1]	0.3 [0.2–0.4]
CV (cm/s)	84.7 [67.6–100.7]	53.5 [31.9–79.2]	85.0 [68.3–100.9]	57.2 [35.0–82.8]
EEA (%)	2.7%	14.2%	2.6%	10.5%

Values are presented as median [interquartile ranges] or incidence (distribution of parameter). Sum of the normal and LVA, values correspond to the total number of cliques (*N* = 164,704) per parameter.

CV, conduction velocity; EEA, endo-epicardial asynchrony; LVA, low-voltage area.

Omnipolar LVAs were present at 11.7% of the endocardium and 4.0% of the epicardium. However, only 22.7% and 30.6% of omnipolar LVAs corresponded to unipolar LVAs at respectively the endo- and epicardial side.

As demonstrated in Table 2, CV was lower and EEA was more pronounced in both unipolar and omnipolar LVAs compared to the non-LVAs. The difference in CV and EEA between LVAs and non-LVAs was smaller in omnipolar LVAs than in unipolar LVAs (*p* < 0.001).

### Prediction of unipolar opposite LVAs

To determine the predictive value of endocardial or epicardial LVAs for the opposite layer, all cliques were subdivided into cliques with either LVA in only one layer (*endo/epi*-LVA) or cliques with LVAs present at both layers (EE-LVA). Characteristics of these clique categories are listed in Table 3. Of all endocardial LVA cliques, 25.8% corresponded to an epicardial LVA and 47.3% of the epicardial LVA cliques corresponded to an endocardial LVA. Therefore, 0.8% of all cliques were identified as EE-LVA, 0.9% as *epi*-LVA and 2.3% as *endo*-LVA. At the opposite site of unipolar *endo-* and *epi*-LVAs, “normal” cliques were characterized by lower V_uni,max_, V_omni,max_, lower CV and enhanced EEA compared to the other non-LVA cliques (*p* < 0.001). Most EEA was found at *endo*-LVA cliques.

**TABLE 3:** Characteristics of low-voltage areas at the opposite side (*N* = 164,704 per parameter).

Endocardial	Unipolar			Omnipolar		
	Normal	*Epi*-LVA	EE-LVA	Normal	*Epi*-LVA	EE-LVA
N (%)	96.0%	0.9%	0.8%	86.3%	1.9%	2.1%
Unipolar (mV)	6.3 [3.4–11.1]	2.2 [1.4–5.0]	0.8 [0.6–0.9]	7.0 [4.2–11.9]	3.2 [1.8–6.2]	1.2 [0.8–1.6]
Omnipolar (mV)	3.5 [1.3–8.9]	1.6 [0.6–4.8]	0.2 [0.2–0.3]	4.2 [1.7–9.8]	2.1 [0.9–6.2]	0.3 [0.2–0.4]
CV (cm/s)	84.4 [63.5–104.0]	68.4 [48.6–89.0]	53.9 [29.2–81.6]	86.7 [66.8–105.4]	70.0 [50.0–91.4]	54.6 [32.7–82.0]
EEA (%)	2.2%	18.3%	9.3%	1.8%	14.0%	7.2%
**Epicardial**	**Normal**	***Endo*-LVA**	**EE-LVA**	**Normal**	***Endo*-LVA**	**EE-LVA**
N (%)	96.0%	2.3%	0.8%	86.3%	9.6%	2.1%
Unipolar (mV)	8.4 [5.2–11.8]	3.8 [2.0–6.5]	8.8 [5.7–12.1]	8.8 [5.7–12.1]	5.6 [3.2–8.8]	1.2 [0.9–1.6]
Omnipolar (mV)	6.9 [3.4–11.0]	3.5 [1.3–7.0]	0.2 [0.2–0.4]	7.1 [3.8–11.3]	5.2 [2.1–9.2]	0.3 [0.2–0.4]
CV (cm/s)	84.8 [67.8–100.8]	78.8 [59.1–98.4]	57.5 [32.7–84.2]	85.7 [69.1–101.4]	78.9 [62.0–95.5]	55.9 [34.2–81.3]
EEA (%)	2.2%	21.3%	9.5%	1.8%	9.2%	7.3%

Values are presented as median [interquartile ranges] or incidence (distribution of parameter).

CV, conduction velocity; EEA, endo-epicardial asynchrony; EE-LVA, endo-epicardial low-voltage area; *Endo*-LVA, endocardial low-voltage area; *Epi*-LVA, epicardial low-voltage area.

### Prediction of omnipolar opposite LVAs

Of all endocardial omnipolar LVA cliques, 18.0% corresponded to an epicardial LVA and 52.4% of the epicardial LVA cliques corresponded to an endocardial LVA. Therefore, 2.1% of all cliques were identified as EE-LVA, 1.9% as *epi*-LVA and 9.6% as *endo*-LVA. At the opposite site of omnipolar *endo-* and *epi*-LVAs, “normal” cliques were characterized by lower V_uni,max_, V_omni,max_, lower CV and enhanced EEA compared to the other non-LVA cliques (*p* < 0.001). Only 7.6% of these cliques corresponded to unipolar LVAs.

### Identification of LVAs by combined unipolar and omnipolar voltage mapping

Cliques containing both unipolar and omnipolar LVAs were identified. Characteristics of these overlapping LVAs are listed in Table 4. At the endocardium, overlapping LVAs consisted of 86.8% of V_uni,max_ and 22.7% of V_omni,max_ compared to 73.7% of V_uni,max_ and 30.6% of V_omni,max_ at the epicardium.

**TABLE 4:** Characteristics of low-voltage areas at the opposite side using combined unipolar and omnipolar voltage mapping (*N* = 164,704 per parameter).

Endocardial	Normal	*Epi*-LVA	EE-LVA
N (%)	96.7%	0.6%	0.6%
Unipolar (mV)	6.3 [3.4–11.1]	2.2 [1.2–5.8]	0.8 [0.6–0.9]
Omnipolar (mV)	3.5 [1.3–8.8]	1.6 [0.5–5.7]	0.2 [0.1–0.3]
CV (cm/s)	84.3 [63.3–103.9]	69.5 [49.2–90.3]	54.6 [29.2–82.6]
EEA (%)	2.4%	20.1%	6.8%
**Epicardial**	**Normal**	***Endo*-LVA**	**EE-LVA**
N (%)	96.7%	2.0%	0.6%
Unipolar (mV)	8.4 [5.1–11.7]	3.9 [2.0–6.6]	0.8 [0.6–0.9]
Omnipolar (mV)	6.8 [3.4–11.0]	3.8 [1.2–7.3]	0.2 [0.2–0.3]
CV (cm/s)	84.7 [67.6–100.7]	79.3 [59.2–98.8]	56.3 [30.6–84.2]
EEA (%)	2.4%	20.9%	6.9%

Values are presented as median [interquartile ranges] or incidence (distribution of parameter).

CV, conduction velocity; EEA, endo-epicardial asynchrony; EE-LVA, endo-epicardial low-voltage area; *Endo*-LVA, endocardial low-voltage area; *Epi*-LVA, epicardial low-voltage area.

In total, 2.6% overlapping endocardial LVAs were identified, compared to 1.2% at the epicardium; 0.6% were identified as EE-LVAs. These EE-LVAs consisted of 23.3% of all endocardial overlapping LVAs and 50.4% at the epicardium. At the opposite site of overlapping *endo-* and *epi*-LVAs, “normal” cliques were characterized by lower V_uni,max_, V_omni,max_, lower CV and enhanced EEA compared to the other non-LVA cliques (*p* < 0.001).

**FIGURE 3:**
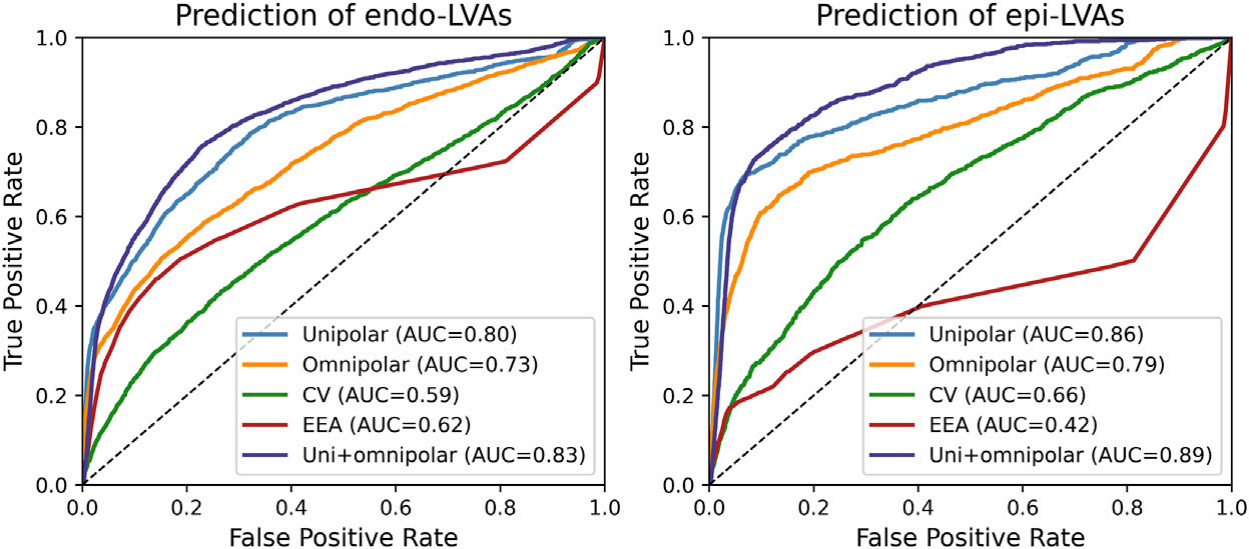
ROC-curves of the prediction of overlapping endocardial (left) and epicardial (right) LVAs based on all parameters recorded from the opposite layer. AUC = area under the curve; CV = conduction velocity; EEA = endo-epicardial asynchrony.

Figure 3 demonstrates ROC-curves of the accuracy of identifying overlapping endocardial and epicardial LVAs based on all parameters recorded at the opposite layer. Combined unipolar and omnipolar voltages were most accurate in identifying overlapping LVAs at the opposite layer (endocardial LVAs: AUC = 0.83; epicardial LVAs: AUC = 0.89).

## Discussion

### Key findings

In most clinical settings, endocardial bipolar voltage mapping is mainly used to identify LVAs, which are considered surrogate markers for areas of diseased tissue. However, in recent perspective, it has become clear that bipolar LVAs can also be located exclusively at the epicardium (Piorkowski et al., 2018). Our study demonstrated that also by using unipolar and omnipolar EGMs, the majority of unipolar and omnipolar LVAs are also located exclusively at either the endocardium or epicardium. More importantly, we have shown that the most accurate methodology to identify EE-LVAs is to combine epicardial unipolar and omnipolar voltages. When using either unipolar or omnipolar voltages separately, epicardial LVAs still corresponded well with endocardial LVAs, but not vice-versa. Within the same layer, only unipolar LVAs corresponded well with omnipolar LVAs. Furthermore, there was a clear relationship between endo-epicardial unipolar voltages, but not between endo-epicardial omnipolar voltages.

### Endo-epicardial voltage mapping

Data on the relationship between atrial endocardial and epicardial voltages is lacking, although it has been studied in the ventricles (Hutchinson et al., 2011; Tokuda et al., 2013). In the atria, van der Does et al. (2018) demonstrated a linear relationship between unipolar endocardial and epicardial voltage in which epicardial voltages were larger than endocardial voltages. Other studies also reported higher mean voltage values in the epicardium compared to mean endocardial voltage, in both atria and ventricles (Lemery et al., 2007; Hutchinson et al., 2011; Tokuda et al., 2013; van der Does et al., 2021). Using our clique voltages, we demonstrated a clear inverse quadratic relation with linear component between endocardial and epicardial unipolar clique voltages. Therefore, unipolar voltages recorded at the endocardium are predictive for epicardial unipolar voltages. In contrast, we found no relationship between endocardial and epicardial omnipolar voltages. This could likely be caused by the limited field-of-view of omnipolar voltages. Consequently, omnipolar voltages recorded at the endocardium are a poor predictor for voltages at the other layer.

### Anatomy of the right atrium

Variations between endocardial and epicardial voltages can also be explained by the anatomical structure of the RA. The surface of the endocardium is very irregular due to the presence of the pectinate muscles and terminal crest, in contrast to the smooth epicardium. Diameters and therefore cardiac mass of the pectinate muscles may vary considerably (Sanchez-Quintana et al., 2002). In addition, the arrangement of pectinate muscles causes variation in the level of contact with the electrodes. Furthermore, due to atrial remodeling and varying wall stress, the atria and therefore the pectinate muscles may elongate resulting in alternation of the bundle density (Schuessler et al., 1993). This results in a non‐uniform spread of activation and can consequently also considerably affect omnipolar voltages. Moreover, Kharbanda et al. (2020) demonstrated that conduction disorders occur more frequently at the endocardium. These features could explain the large discrepancies in unipolar and omnipolar voltages at endocardial LVAs. As the epicardial surface is more smooth, proper tissue contact with the entire electrode array can be achieved more easily. Therefore, epicardial LVAs could be more likely indicative of structural abnormalities and hence EE-LVAs.

### Identification of endo-epicardial substrate

The arrhythmogenic substrate can be located either subendocardially, subepicardially, intramurally or transmurally (Tokuda et al., 2013; Chen et al., 2018; Gharaviri et al., 2020). Piorkowski et al. (2018) recently demonstrated by bipolar endo-epicardial mapping in the left and RA that LVAs were present in 44 patients with history of AF in either both layers (*N* = 33, 75%) or in solely the endocardium (*N* = 6, 14%) or epicardium (*N* = 6, 14%). However, all patients already had (multiple) ablation procedures. Our study population consisted of patients without previous ablation therapy and we demonstrated that the majority of the LVAs at the RA were exclusively located at either the endocardium or epicardium. These findings demonstrate that the arrhythmogenic substrates may not necessarily be transmural present. Verheule et al. (2013) also demonstrated in a goat model of AF that formation of endomysial fibrosis located within the outer millimeter of the epicardial layer accompanied the transition from persistent to permanent AF, while endocardial bundles remained unaffected. Hence, as structural remodeling can occur locally within one layer, the presence of a LVA can also be confined to either the endocardial or epicardial layer. This could in turn lead to increased EEA due to progressive uncoupling between the epicardial layer and the endocardial bundles, which also has been proposed as an important mechanism for AF (de Groot et al., 2016).

On the other hand, as omnipolar voltages represent more local activity, this recording modality is more likely to only detect LVAs representing substrate located either (sub) endocardially or (sub) epicardially depending on the recording location. Several studies have shown an added value of combining unipolar and bipolar voltages to detect intramural or transmural substrates (Spears et al., 2012; Casella et al., 2015; Yalin et al., 2015). Hutchinson et al. (2011) demonstrated that epicardial bipolar LVAs can be identified in most patients with left ventricular cardiomyopathy using endocardial unipolar voltage mapping. However, Tokuda et al. (2013) observed discrepancies in the accuracy of epicardial bipolar LVA identification using endocardial unipolar voltages between patients with nonischemic and ischemic cardiomyopathy. In our present study, we demonstrated that endocardial LVAs at the atria frequently did not correspond to LVAs at the epicardium, independently of the recording technique used. Even by combining unipolar and omnipolar voltages, only 23.3% of the endocardial LVAs corresponded to epicardial LVAs, favoring a combined endocardial and epicardial mapping approach.

### Endo-epicardial ablation therapy

Especially in patients with persistent AF, the presence of complex 3-dimensional arrhythmogenic substrates limits the efficacy of endocardial ablation strategies. Minimally invasive surgical ablation of AF is therefore increasingly combined with the endocardial transcatheter procedures in so-called hybrid procedures, showing promising results (Edgerton et al., 2016; Piorkowski et al., 2018; De Martino et al., 2021; Zheng et al., 2021). In the study of Piorkowski et al. (2018), 73% of complex AF patients remained free from AF during 2 years of follow-up, although other studies encountered higher recurrence rates (Gehi et al., 2013; Edgerton et al., 2016; Jiang et al., 2019). However, LVAs frequently occur at either the endocardium or epicardium alone and the opposite areas could contain normal voltages. These areas are therefore “invisible” when recording from only one side. Diseased tissue could then consequently be missed using a one-sided approach. In addition, epicardial mapping provides more often accurate identification of EE-LVAs and the role of epicardial structures, such as Bachmann’s bundle, become more recognized in the pathogenesis of AF (Teuwen et al., 2016; De Martino et al., 2021). This suggests that a strategy of combined endo-epicardial access for mapping and ablation may provide superior efficacy to an endocardial-only approach.

### Study limitations

Intraoperative simultaneous endo-epicardial mapping in humans can only be safely performed during cardiac surgical procedures. Therefore, as endo-epicardial mapping was only performed on a limited area of the RA free wall, we could not evaluate the relation of endocardial and epicardial voltages in the complete atria, specifically not in the left atrium. The recorded potentials might be influenced by the presence of epicardial fat as previous studies demonstrated that the presence of thick epicardial fat is associated with attenuated bipolar voltage (Saba et al., 2009; Desjardins et al., 2010). Although we did not experience any large effects of visually present epicardial fat at the RA, we cannot ascertain that the presence of epicardial fat has influenced our results. In addition, the underlying anatomy such as variability in atrial wall thickness and the degree of trabeculation cannot be retrieved, and exact tissue histology could not be performed. Therefore, we were unable to correlate the exact underlying anatomy and histology with the mapping data. This study focused on the comparison of the different voltage mapping methodologies and identification of LVAs without interventions. The next step will be to incorporate the results of this study with ablation targeting EE-LVAs in order to determine whether the combination of low unipolar and low omnipolar voltage can improve ablation outcomes.

## Conclusion

When using unipolar and omnipolar EGMs, LVAs are frequently located exclusively at either the endocardium or epicardium and could be undetectable when measuring from the opposite layer only. An endo-epicardial mapping approach is therefore favored to accurately identify LVAs. EE-LVAs are most accurately identified using combined epicardial unipolar and omnipolar voltages. Therefore, a combination of simultaneously recorded endo-epicardial low unipolar *and* low omnipolar voltage may be more indicative of dual-layer LVAs and probably arrhythmogenic substrates.

## Data Availability

The datasets presented in this article are not readily available because of EU privacy law. Requests to access the datasets should be directed to NG, n.m.s.degroot@erasmusmc.nl.
